# Autoimmune encephalitis: Early and late findings on serial MR imaging and correlation to treatment timepoints

**DOI:** 10.1016/j.ejro.2024.100552

**Published:** 2024-02-02

**Authors:** Mahmoud Abunada, Nathalie Nierobisch, Riccardo Ludovichetti, Cyril Simmen, Robert Terziev, Claudio Togni, Lars Michels, Zsolt Kulcsar, Nicolin Hainc

**Affiliations:** a Department of Neuroradiology, Clinical Neuroscience Center, University Hospital Zurich, University of Zurich, Switzerland; b Department of Neurology, Clinical Neuroscience Center, University Hospital Zurich, University of Zurich, Switzerland

**Keywords:** Autoimmune diseases, Magnetic resonance imaging, Encephalitis, Brain

## Abstract

**Introduction:**

MRI is negative in a large percentage of autoimmune encephalitis cases or lacks findings specific to an antibody. Even rarer is literature correlating the evolution of imaging findings with treatment timepoints. We aim to characterize imaging findings in autoimmune encephalitis at presentation and on follow up correlated with treatment timepoints for this rare disease.

**Methods:**

A full-text radiological information system search was performed for “autoimmune encephalitis” between January 2012 and June 2022. Patients with laboratory-identified autoantibodies were included. MRI findings were assessed in correlation to treatment timepoints by two readers in consensus. For statistical analysis, cell-surface vs intracellular antibody groups were assessed for the presence of early limbic, early extralimbic, late limbic, and late extralimbic findings using the χ^2^ test.

**Results:**

Thirty-seven patients (female n = 18, median age 58.8 years; range 25.7 to 82.7 years) with 15 different autoantibodies were included in the study. Twenty-three (62%) patients were MRI-negative at time of presentation; 5 of these developed MRI findings on short-term follow up. Of the 19 patients with early MRI findings, 9 (47%) demonstrated improvement upon treatment initiation (7/9 cell-surface group). There was a significant difference (p = 0.046) between the MRI spectrum of cell-surface vs intracellular antibody syndromes as cell-surface antibody syndromes demonstrated more early classic findings of limbic encephalitis and intracellular antibody syndromes demonstrated more late extralimbic abnormalities.

**Conclusion:**

MRI can be used to help narrow the differential diagnosis in autoimmune encephalitis and can be used as a monitoring tool for certain subtypes of this rare disease.

## Highlights

• MRI can be used as a monitoring tool for certain subtypes of autoimmune encephalitis.
• We found significant differences in the MRI findings of cell-surface compared to intracellular auto-antibodies.
• Almost half of patients with initial/subacute findings demonstrated MRI improvement upon initiation of treatment.

## 1 Introduction

Autoimmune encephalitis is a rapidly expanding medical field driven by an unprecedented antibody discovery rate and improving laboratory diagnostics [1]. Once thought to be exceedingly rare, a recent study found the incidence and prevalence of autoimmune encephalitis to be on par with that of infectious encephalitis [2]. The discovery rate of 1 to 2 new antibodies per year combined with increasing clinical awareness and subsequent initiation of diagnostics is largely responsible for this uptrend [1]. Diagnosis is often delayed, as the clinical phenotype can span the entire spectrum of neurological findings [3] potentially leading to misallocation of symptoms to other neurological or psychiatric disorders [4], with further differential diagnoses including tumors, prion disease, metabolic disorders, and infectious encephalitidies. In fact, the mean delay from symptom onset to antibody testing at a large European referral center was found to be 74 days in 2016, which is a drastic improvement over the 483 day time period found in 2012 [4]. Still, this study highlights the need for improvement, as early diagnosis and initiation of treatment can lead to improved outcomes with reduced disability in patients with autoimmune encephalitis [5], [6].

MRI is performed early upon patient presentation, however can be negative in a large percentage of cases (17 to 89%) [7] or lack findings specific to an antibody. It is unknown how many MRI negative patients go on to develop MRI abnormalities over time. Furthermore, literature correlating the evolution of imaging findings with treatment timepoints is scarce. Thus, the goals of this study are manifold. First, we aim to characterize the spectrum of imaging findings and determine the negative rate on MRI performed upon patient presentation at our tertiary referral center. Second, we aim to quantify the number of initially MRI negative patients that develop MRI findings either on short-term or long-term follow up. Finally, we correlate treatment timepoints to changes on MRI adding to the body of knowledge on follow-up imaging of autoimmune encephalitis.

## 2 Methods

### 2.1 Patient cohort

Approval by the local ethics committee was obtained prior to commencing the study (Kantonale Ethikkommission Zuerich, BASEC Nr. 2022-00041). Informed consent was obtained for all patients. A full-text radiological information system search was performed for the term “autoimmune encephalitis” between January 2012 and June 2022. Patients with autoantibody-positive encephalitis were included. Patients with the diagnosis of seronegative autoimmune encephalitis were excluded.

**Fig. 1:**
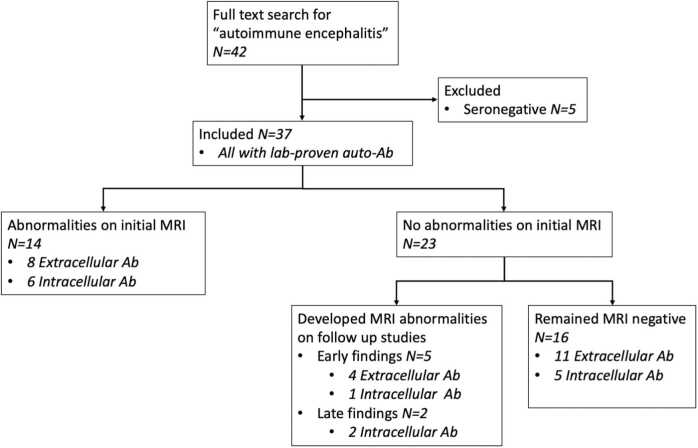
Flowchart of selection process: Autoimmune encephalitis cohort with inclusion and exclusion criteria. Ab=Antibody.

37 patients (female n = 18, median age 58.8 years; range 25.7 to 82.7 years) were included in the study (Fig. 1), all with antibody-proven autoimmune encephalitis (4 anti-NMDAR, 2 anti-GABAaR, 2 anti-GABAbR, 5 anti-LGI1, 4 anti-CASPR2, 2 anti-VGKC, 1 anti-VGCC, 2 anti-IgLON5, 1 anti-GFAP, 3 anti-Hu, 1 anti-Ri, 3 anti-Yo, 1 anti-Ma2/Ta, 1 anti-CV2, 5 anti-GAD).

### 2.2 MRI findings

MR imaging of the brain was routinely performed with administration of intravenous contrast and the entire protocol consisted of axial contrast-enhanced FLAIR, T2, DWI, SWI, and 3D T1 MPRAGE pre- and post-contrast, as per institutional protocol. Patients were grouped according to laboratory diagnosis into either cell-surface or intracellular autoantibody (Table 1). MRI findings on initial presentation and follow-up exams were assessed in correlation to treatment timepoints. MRI Reading was performed in consensus by two neuroradiologists (MA and NH) with 4 and 9 years of neuroradiology reading experience. For statistical analysis, all patients were assessed for the presence of early limbic, early extralimbic, late limbic, and late extralimbic findings. Early was defined as FLAIR hyperintense signal change (or other characteristic MRI findings of autoimmune encephalitis) present on either initial MRI or on short-term MRI follow-up studies. Late was defined as persistent signal change on final MRI or progressive volume loss (atrophy).

**Table 1:** Autoantibody targets included in the cohort separated into cell-surface and intracellular groups.

Cell-surface antibodies	Intracellular antibodies
NMDAR	Hu
GABAaR	Ri
GABAbR	Yo
LGI1	Ma2/Ta
CASPR2	CV2
VGKC	GAD
VGCC	
IgLON5	
GFAP	

### 2.3 Statistical analysis

The distribution of imaging findings between the cell-surface group and the intracellular group was assessed using the χ^2^ test. Significance was set to p < .05.

## 3 Results

### 3.1 Patient cohort

**Fig. 2:**
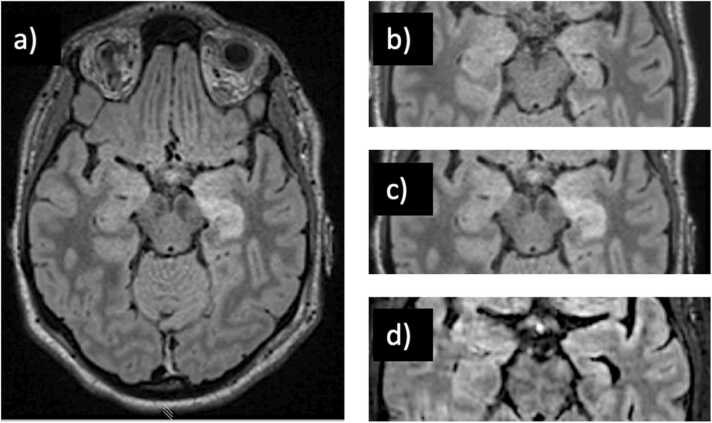
A patient with anti-NMDA receptor encephalitis (a) Whole-brain FLAIR image demonstrating hyperintense signal change and enlargement of the left hippocampus. This finding was not seen on initial MRI (b). (c) Cropped FLAIR image of MRI performed on day 5 for comparative purposes. (d) Improvement of findings upon initiation of corticosteroids and plasmapheresis with development of left hippocampus atrophy on long-term follow-up.

**Fig. 3:**
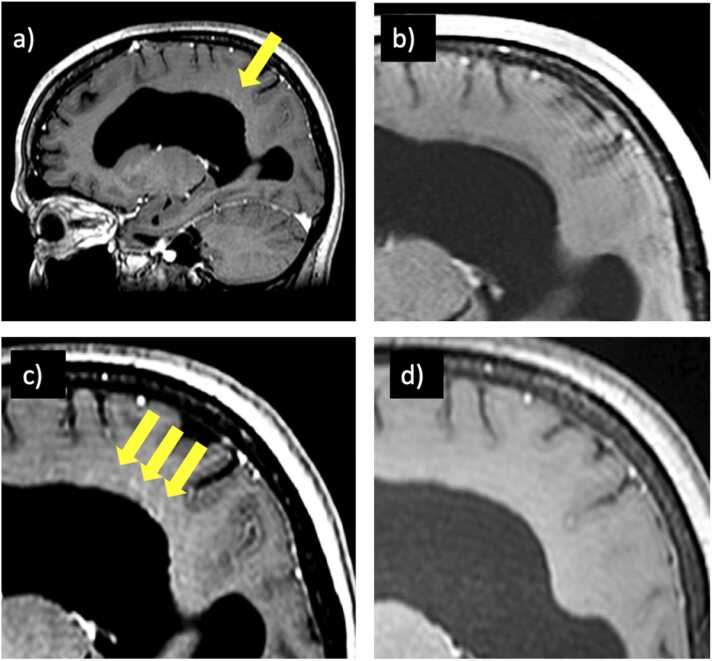
A patient with anti-GFAP autoimmune encephalitis. (a) Whole-brain sagittal contrast-enhanced T1 image demonstrating linear strands of contrast enhancement radiating outwards from the ventricle. This finding was not seen on initial MRI (b). (c) Cropped, zoomed image of (a) performed on day 95 where the finding was first noted. (d) Resolution of findings upon second-line treatment with rituximab.

**Fig. 4:**
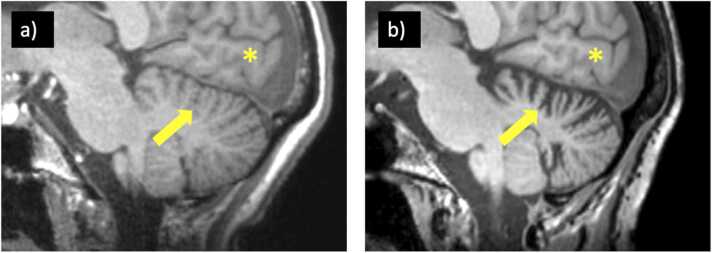
A patient with anti-GAD encephalitis. Sagittal T1 weighted images performed on patient presentation (a) and 3 years later (b) demonstrating interval development of isolated cerebellar atrophy (arrows in a and b) compared with the occipital lobe (star) which did not develop atrophy.

23 of 37 (62%) patients presented with negative MRI studies, and 7 of these 23 eventually developed MRI findings on follow-up studies (anti-NMDAR, 2 anti-LGI1, anti-GFAP, anti-Ma2/Ta, anti-GAD, anti-Hu). 5 of 7 eventually developed MRI abnormalities in the acute/subacute setting (mean 56 days, SD 40 days) including FLAIR hyperintense signal change of the hippocampus, amygdala, or both (Fig. 2). One patient (anti-GFAP) developed radial contrast enhancement (Fig. 3). 2 of the 7 initially MRI negative patients developed long-term findings including basal ganglia atrophy (306 days) and cerebellar atrophy (3.2 years; Fig. 4). 36 of 37 patients had at least one follow-up MRI study with a mean follow-up time of 2.1 ± 1.9 years, performed upon requisition by the primary care physician. A summary of imaging findings can be found in Table 2 (cell-surface antibodies) and Table 3 (intracellular antibodies).

**Table 2:** Summary of imaging findings for cell-surface antibodies.

Entity	Patient	Initial MRI Abnormality	Short term development of findings?	Treatment	Long term finding
Anti-NMDAR	1	None	Left hippocampus	Corticosteroids, plasmapheresis	Left hippocampus atrophy
	2	None	No	Corticosteroids, IVIG	None
	3	None	No	Corticosteroids, IVIG	None
	4	Right hippocampus		IVIG, Corticosteroids, plasmapheresis, cyclophosphamide	Right hippocampus atrophy
Anti-GABAaR	1	Multifocal, asymmetrical FLAIR hyperintense lesions involving the cortex and subcortical white matter. Both hippocampi involved		Corticosteroids, plasmapheresis, cyclophosphamide, rituximab	Bilateral hippocampus atrophy
	2	Multifocal, asymmetrical FLAIR hyperintense lesions involving the cortex and subcortical white matter (including cingulate gyrus).		Corticosteroids, plasmapheresis	Bilateral hippocampus atrophy
Anti-GABAbR	1	None	No	None	None
	2	Left hippocampus		Corticosteroids, IVIG, chemotherapy	Bilateral hippocampus signal change
Anti-LGI1	1	None	No	Corticosteroids	None
	2	None	Left hippocampus and amygdala	Corticosteroids, plasmapheresis, rituximab	Left hippocampus atrophy
	3	Both hippocampi		IVIG, Corticosteroids, rituximab	Bilateral hippocampus atrophy
	4	None	Left amygdala	None	None
	5	Left hippocampus, both amygdalae		Corticosteroids, IVIG	Left hippocampus atrophy and bilateral amygdala signal change
Anti-CASPR2	1	None	No	Corticosteroids, rituximab	None
	2	None	No	Plasmapheresis, IVIG, corticosteroids, rituximab	None
	3	Both hippocampi	Bilateral claustrum signal change	Corticosteroids, IVIG, tumor surgery, radiotherapy	Bilateral claustrum signal change
	4	None	No	None	None
Anti-VGKC	1	None	No	Rituximab	None
	2	None	No	Corticosteroids, plasmapheresis	None
Anti-VGCC	1	None	No	None	None
Anti-IgLON5	1	None	No	Corticosteroids, plasmapheresis, rituximab	None
	2	Pons		None	Pons signal change
Anti-GFAP	1	None	Linear strands of contrast enhancement radiating outwards from ventricles	Corticosteroids, rituximab	None
Summary cell-surface			Early limbic n = 10		Late limbic n = 8
			Early extralimbic n = 5		Late extralimbic n = 2

**Table 3:** Summary of imaging findings for intracellular antibodies.

Entity	Patient	Initial MRI Abnormality	Short term development of findings?	Treatment	Long term finding
Anti-Hu	1	None	No	Corticosteroids, IVIG, rituximab	Right basal ganglia atrophy
	2	Pons and left pulvinar	No	IVIG	Pons signal change
	3	None	No	IVIG, rituximab, cyclophosphamide	None
Anti-Ri	1	None	No	None	None
Anti-Yo	1	Cerebellum	No	Surgery, IVIG	Cerebellar atrophy
	2	Putamen bilaterally	No	Chemotherapy, corticosteroids	Putaminal fork sign bilaterally
	3	None	No	Corticosteroids, IVIG, rituximab, plasmapheresis	None
Anti-Ma2/Ta	1	None	Multifocal, asymmetrical FLAIR hyperintense lesions involving the cortex and subcortical white matter pronounced in the temporal lobe.	Corticosteroids	Hippocampal and temporal lobe signal change
Anti-CV2	1	None	No	Chemotherapy	None
Anti-GAD	1	None	No	Corticosteroids, rituximab	Cerebellar atrophy
	2	None	No	None	None
	3	Hippocampi bilaterally and cerebellum		IVIG, Corticosteroids	Hippocampal and cerebellar signal change
	4	Left hippocampus and amygdala		None	Left hippocampus atrophy
	5	Vermis	No	Corticosteroids, rituximab	Vermian atrophy
Summary Intracellular			Early limbic n = 2		Late limbic n = 3
			Early extralimbic n = 5		Late extralimbic n = 6

**Fig. 5:**
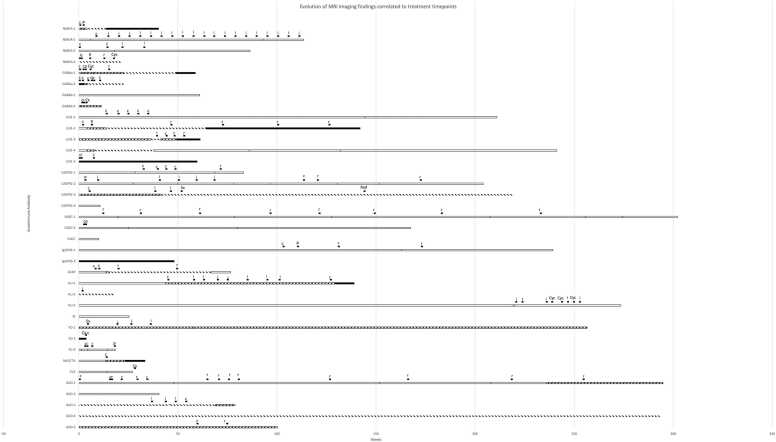
Bar graph of all patients included arranged by autoantibody including treatment timepoints. White bar = no MRI abnormalities. Black bar = MRI abnormalities (stable). Upwards diagonal lines = progressive MRI abnormalities. Downwards diagonal lines = improving MRI abnormalities. Treatment: c = corticosteroids; p = plasmapheresis; i = IVIG; r = Rituximab; Cyc = Cyclophosphamide; Cx = chemotherapy; Sx = surgery; Rad= radiation therapy.

Treatment information was available for all patients, and 30 patients received treatment over their respective MRI follow-up intervals (Fig. 5): first-line immunotherapy (corticosteroids, plasma exchange, intravenous immunoglobulins [IVIG]) was administered in 27 patients, second-line immunotherapy (rituximab or cyclophosphamide) was administered in 15 patients. Three patients underwent chemotherapy for underlying malignancies. Two further patients underwent surgery (one with adjuvant radiotherapy) for an underlying malignancy.

**Fig. 6:**
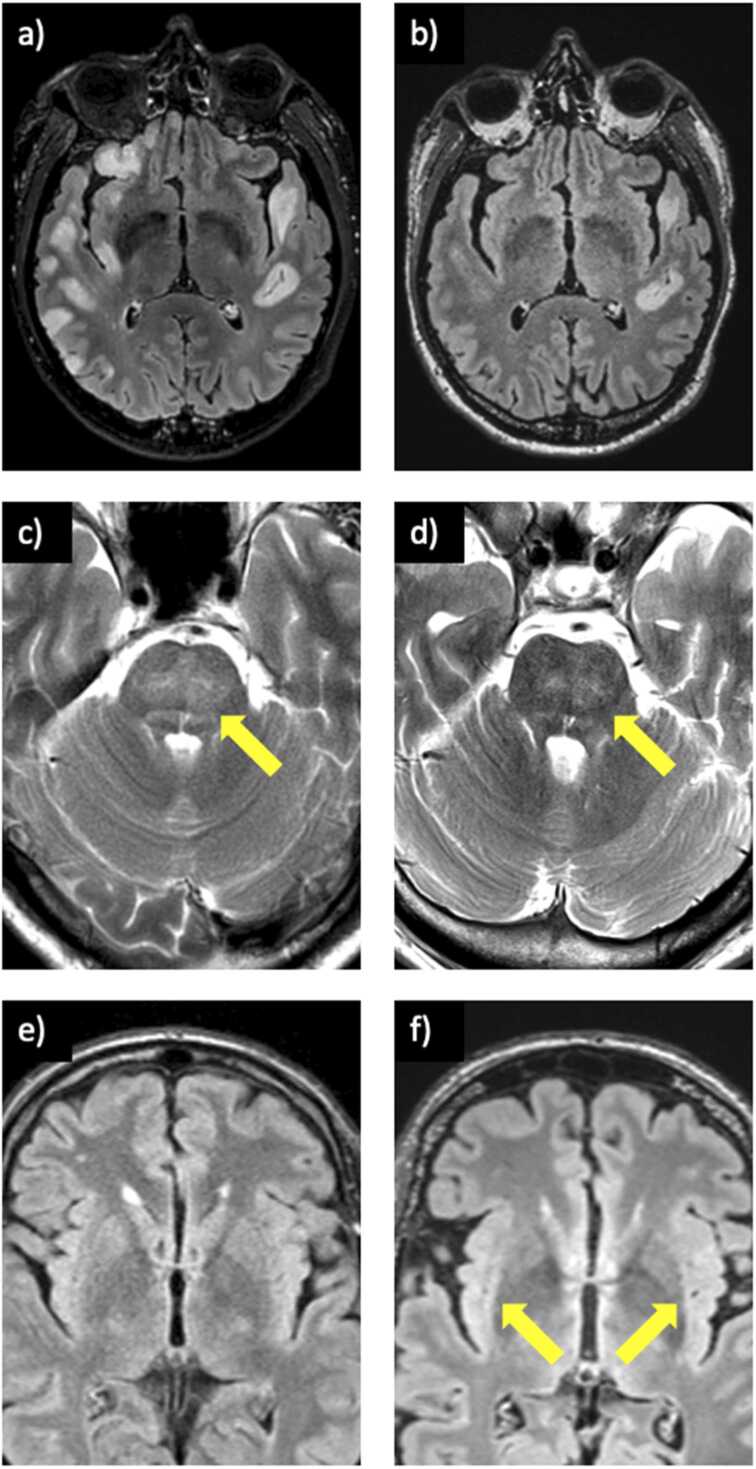
Further examples of autoimmune encephalitis with follow up imaging. (a) A patient with anti-GABAaR encephalitis demonstrating multifocal, asymmetrical FLAIR hyperintense lesions involving the cortex and subcortical white matter on initial MRI; (b) marked improvement on MRI at day 156 after treatment with corticosteroids and plasma exchange. (c) A patient with anti-Hu encephalitis demonstrating signal change within the pons (arrow); (d) follow-up MRI on day 141 after treatment with plasma exchange and IVIG showed improvement but not complete resolution of findings (arrow). (e) A patient with anti-CASPR2 encephalitis with a bilaterally normal-appearing claustrum on initial MRI; (f) MRI performed four years later reveals new signal change in the claustrum bilaterally (arrows) despite treatment with corticosteroids and IVIG.

Of the 19 patients presenting with abnormalities on initial MRI (14) and the 5 that developed early MRI findings on short-term follow up, 9 (47%) demonstrated improvement upon treatment initiation. This includes anti-NMDAR (2; both after first-line treatment), anti-GABAaR (2; 1 after first line, one after second-line treatment; Fig. 6), anti-LGI1 (1; after combined first and second-line treatment together), anti-CASPR2 (1; after first-line treatment), anti-GFAP (1; after second-line treatment), anti-Hu (1; after first-line treatment), and anti-GAD (1; after first-line treatment).

### 3.2 MRI findings

#### 3.2.1 Anti-NMDAR

3 of 4 patients presenting with anti-NMDA receptor encephalitis presented with negative MRI at presentation, with one of these becoming positive on follow-up MRI on day 5 (Patient NMDA-1). Imaging findings were limited to FLAIR hyperintense, enlarged hippocampi (unilateral in both cases) which atrophied on follow-up imaging; acute hippocampal findings in both patients regressed after treatment which consisted of corticosteroids and plasmapheresis (both patients) and IVIG in one patient (Patient NMDA-4). The other 2 patients remained negative throughout all follow-ups.

#### 3.2.2 Anti-GABAaR

2 of 2 patients demonstrated multifocal, asymmetrical FLAIR hyperintense lesions involving the cortex and subcortical white matter bilaterally which progressed despite corticosteroid treatment. Imaging findings in Patient GABAa-2 eventually decreased upon plasmapheresis while Patient GABAa-1′s findings decreased upon Rituximab and Cyclophosphamide administration (after initial corticosteroids and plasmapheresis). The hippocampus was only involved in 1 patient initially with gradual hippocampal involvement in the second patient over time. A small area of leptomeningeal contrast enhancement developed and resolved in one patient over the insular cortex. Both patients demonstrated FLAIR hyperintense, atrophied hippocampi on final MRI.

#### 3.2.3 Anti-GABAbR

Only 1 of 2 patients demonstrated imaging findings, initially with FLAIR hyperintense signal change of one hippocampus, evolving to both over time (no enlargement over all timepoints; Patient GABAb-2). Hippocampal findings remained unchanged despite treatment with corticosteroids, IVIG, and chemotherapy for small cell lung cancer.

#### 3.2.4 Anti-LGI-1

3 of 5 patients presented with unremarkable MRI studies, with 2 of these evolving to demonstrate findings on day 29 (LGI1–2; unilateral enlargement and FLAIR hyperintensity of the hippocampus and amygdala) and day 55 (LGI1–4; unilateral enlargement and FLAIR hyperintensity of the amygdala). Patient LGI1–2 demonstrated normalization of the initial enlargement with gradual volume loss of the hippocampus on final MRI. MRI findings in LGI1–4 regressed completely without documented treatment over the entire imaging interval.

The 2 patients with MRI findings at presentation both demonstrated FLAIR hyperintense hippocampi, one patient with bilateral hippocampal enlargement (LGI1–3; which evolved to atrophy after IVIG, corticosteroids, and rituximab), the other with unilateral hippocampal atrophy and bilateral enlargement and signal change of the amygdala (LGI1–5; which did not change after IVIG and corticosteroids).

#### 3.2.5 Anti-CASPR2

Only 1 of 4 patients with anti-CASPR2 antibodies demonstrated imaging findings, initially presenting with FLAIR hyperintense signal change of the hippocampi bilaterally. Findings progressed to include bilateral FLAIR signal change of the claustrum despite corticosteroid treatment. Upon administration of IVIG, findings began to decrease with normalization of the hippocampi but persistent claustrum findings.

#### 3.2.6 Anti-VGKC

Both patients presented with unremarkable MRI studies over all follow-ups.

#### 3.2.7 Anti-VGCC

The patient presented with unremarkable MRI studies over all follow-ups.

#### 3.2.8 Anti-IgLON5

1 of 2 patients presented with FLAIR hyperintense signal change of the pons on initial MRI which did not change over the duration of follow up studies.

#### 3.2.9 Anti-GFAP

The patient with anti-GFAP presented with an unremarkable MRI study which evolved to tiny linear strands of contrast enhancement radiating outwards through the hemispheric white matter from the lateral ventricles despite corticosteroid treatment. This finding disappeared after initiation of rituximab.

#### 3.2.10 Anti-Hu

Only 1 of 3 patients presented with imaging findings (HU-2; FLAIR hyperintense signal change within the pons and left pulvinar) which decreased upon IVIG administration, with another patient developing findings at 44 weeks follow up (HU-1; bilateral basal ganglia atrophy with gliosis and hemosiderin deposition) despite treatment with corticosteroids, IVIG, and rituximab.

#### 3.2.11 Anti-Ri

The patient presented with unremarkable MRI studies over all follow ups.

#### 3.2.12 Anti-Yo

2 of 3 patients demonstrated imaging findings upon presentation: YO-1 with progressive cerebellar atrophy despite corticosteroids, IVIG, and rituximab, and YO-2 with FLAIR hyperintense putaminal fork signs that persisted on final MRI. YO-2′s treatment with corticosteroids and chemotherapy for metastasized ovarian cancer was initiated simultaneously with the final MRI. Patient YO-3 did not demonstrate MRI abnormalities.

#### 3.2.13 Anti-Ma2/Ta

The patient evolved from presenting with an unremarkable MRI to demonstrating bilateral temporal cortical and subcortical FLAIR hyperintense signal change including the hippocampus despite treatment with corticosteroids.

#### 3.2.14 Anti-CV2

The patient presented with unremarkable MRI studies over all follow ups.

#### 3.2.15 Anti-GAD

2 of 5 patients presented with unremarkable MRI studies, with one demonstrating cerebellar atrophy at 4 years of follow up (GAD-1) after multiple courses of corticosteroids and rituximab treatment. Patient GAD-5 had slight vermian atrophy on initial MRI which progressed on 2 year follow-up. Patient GAD-4 presented with bilaterally enlarged and FLAIR hyperintense hippocampi which became atrophic on long-term follow up (no documented treatment). Finally, Patient GAD-3 initially demonstrated bilaterally enlarged and FLAIR hyperintense hippocampi along with multiple ill defined, patchy contrast-enhancing FLAIR hyperintensities of the cerebellum. These findings decreased under IVIG and corticosteroids, however, they progressed up to the final MRI, with no treatment documented in the progressive interval.

### 3.3 Statistical analysis

The χ^2^ test revealed a significant difference in MRI findings between the cell-surface and intracellular groups (p = 0.046).

## 4 Discussion

In our autoimmune encephalitis cohort of 37 patients imaged over 10 years, only 14 (38%) demonstrated abnormalities on initial MRI (i.e. a negative rate of 62%) with a further 5 (14%) developing findings in the subacute setting. This is comparable to a recent large cohort study by Gillon et al. involving 85 patients over a 10-year time period where 52 (61%) demonstrated MRI abnormalities upon symptom onset [8]. Further corroborating the findings of Gillon et al., we found a significant difference in the MRI presentation of cell-surface vs intracellular autoantibody syndromes, where the cell-surface group demonstrated more acute and classic findings of limbic encephalitis including signal change of the hippocampus whereas the intracellular group demonstrated long-term cerebellar degeneration more often. These differences correlate to what is expected in the literature, as antibodies targeting intracellular neuronal antigens more often demonstrate irreversible neuronal damage leading to cerebellar degeneration [9]. With cell-surface antibodies, neurologic outcomes are more closely related to treatment administration, i.e. clinical improvement upon immunosuppression [10], [11]; thus, a similar course is expected for MRI abnormalities. In our cohort, 9 of 19 patients with MRI abnormalities on initial imaging or in the subacute setting demonstrated improvement upon initiation of treatment. Correspondingly, the majority (7/9) of these were cell-surface antibody syndromes. The following is a literature review of all entities included in our study with a focus on acute and long-term MRI abnormalities.

### 4.1 Anti-NMDAR

First discovered in 2007, anti-NMDAR encephalitis is a disease predominantly affecting young women with a remarkably high rate of unremarkable MRI; sensitivities range from 11 to 83% [1], [7]. A recent review article incorporating 56 MRI imaging articles reported a mean rate of abnormal MRI of 37.7% in the acute phase of anti-NMDA encephalitis [7]. A wide range of MRI findings have been reported and Zhang et al. have recently grouped these into 4 different types: Type, 1 normal MRI; Type 2, only hippocampal lesions; Type 3, lesions not involving the hippocampus; and Type 4, lesions involving both the hippocampus and extrahippocampal structures. Type 1 was by far the most common pattern with 53%, followed by Type 4 (44%), and Type 2 and 3 (28% each), with lesions described to involve the cingulate gyrus, corpus callosum, insula, basal ganglia, thalamus, frontal, temporal, and occipital lobes, internal capsule, brain stem, and middle cerebellar peduncle. Pronounced brain atrophy on long-term follow ups (5 to 7 years) has also been described.

### 4.2 Anti-GABAaR

Anti-GABAaR encephalitis is an exceedingly rare entity and MRI sensitivity is reported to be on the order of 77% [12]. A publication from 2020 cites only 50 cases since the first report in 2014, with 83% demonstrating abnormalities on MRI [13]. When positive, a dichotomous phenotype classification has been proposed, separating cases into either confluent or spotted type using a lesion size cutoff of 3 cm [14]. Frontal, temporal, and limbic areas were involved and all patients had cingulate gyrus involvement. Confluent type was associated with more severe disease. Cerebellum and brainstem lesions are rare but have been reported [12].

With regards to follow-up, a dynamic evolution of brain lesions has been described, with some lesions diminishing and others enlarging at 6 months [14]. Long-term follow-up was described to result in atrophy.

### 4.3 Anti-GABAbR

The majority of MRI findings in anti-GABAbR encephalitis are concentrated on the hippocampus and amygdala with initial T2/FLAIR hyperintense signal change and long-term atrophy including hippocampal sclerosis [15] [16]. A systematic review incorporating 94 patients over 10 studies found that 51 (54.8%) had either unilateral or bilateral temporal lobe hyperintensity in the acute setting [17]. In addition to the hippocampus, the predominance of GABAb receptors in the thalamus and brainstem has resulted in rare imaging findings involving some these areas [18] with further rare findings reported in the frontal and temporal lobes [19].

### 4.4 Anti-LGI1

Anti-LGI1 encephalitis also presents with classic MRI findings of limbic encephalitis including T2/FLAIR hyperintense signal change of the hippocampus and amygdala followed by hippocampal atrophy on long-term follow-up [15]. First reported in 2010 [20], anti-LGI1 encephalitis is the second most common autoimmune encephalitis after anti-NMDAR encephalitis and the most common etiology of limbic encephalitis [21], [22]. In a recent study involving 76 patients, 57 (57%) were described as MRI positive with predominant hippocampal (89%) and basal ganglia (28%) involvement [23]; long-term basal ganglia atrophy [23], whole-brain atrophy [15], and hippocampal sclerosis [24] have also been described. Supratentorial white matter blurring has been reported in a small number of cases [25].

### 4.5 Anti-CASPR2

Anti-CASPR2, like anti-LGI1, is also directed against a subunit of the VGKC complex and is associated with preferential involvement of the medial temporal lobes, although MRI sensitivities are much lower [15]. A recent study involving 38 patients with anti-CASPR2 encephalitis reported an MRI sensitivity of only 30% with most findings pertaining to the hippocampus (24%) and single patients with brainstem and cerebellar involvement. Furthermore, unlike anti-LGI1, hippocampal atrophy/sclerosis is not generally seen on long-term follow-up [24], [26], however, supratentorial white matter blurring has been reported, again at a lower rate [25].

### 4.6 Anti-VGKC

There are no specific imaging findings to differentiate this entity from the anti-LGI1 and anti-CASPR2 syndromes.

### 4.7 Anti-VGCC

Literature on anti-VGCC encephalitis imaging is sparse and limited to case reports and small numbers in cohort studies. When positive on MRI, migratory cortical/subcortical lesions with associated contrast enhancement and cortical laminar necrosis on follow-up studies have been described [9], [27]. Isolated hippocampal involvement has also been shown [28]. With respect to longer-term sequelae, the anti-VGCC antibody has been implicated in cerebellar degeneration [29]. Typically, anti-VGCC is associated with Lambert-Eaton myasthenic syndrome, a disorder of the peripheral nervous system.

### 4.8 Anti-IgLON5

Literature on anti-IgLON5 encephalitis imaging is also very limited. A recent study involving 22 patients describes negative MRI in 18 (81.8%) cases [30]. When positive, brainstem and hippocampal atrophy were noted in keeping with long-term changes. A case report on a patient imaged 3 days after presentation demonstrated symmetric restricted diffusion in the thalamus, brainstem, and cerebellar hemispheres [31]. A second case report in the acute setting demonstrated asymmetrical areas of signal abnormality in the right occipital lobe and left tegmentum [32].

### 4.9 Anti-GFAP

Anti-GFAP was only recently discovered in 2016 [33] and demonstrates a hallmark finding of perivascular radial contrast enhancement on MRI [34]. More recently, a study including 39 patients added to this phenotype to include periventricular T2/FLAIR hyperintense signal change also including the brainstem, thalamus, internal capsule, basal ganglia, and limbic system; thus, the phenotype varies greatly. Of note, 4 of 39 patients demonstrated a mild encephalopathy/encephalitis with reversible splenial lesions [35].

### 4.10 Anti-Hu

Anti-Hu is the most common form of paraneoplastic (i.e. cancer-associated) autoimmune encephalitis (75% with small cell lung cancer) and can result in widespread, variable involvement of the central and peripheral nervous system, which is also reflected on MRI [9], [36], [37]. Findings range from classic limbic encephalitis to encephalomyelitis involving the basal ganglia, brainstem, cerebellum [38], and cranial [39] and spinal nerves [40], [41]. Autonomic nervous system involvement has been described in the context of encephalomyelitis [38]. Long-term sequelae include cerebellar degeneration [37].

### 4.11 Anti-Ri

Anti-Ri is associated with breast and small-cell lung cancer and demonstrates findings of brainstem encephalitis in the acute stage including involvement of the globus pallidus and internal capsule [42] with cerebellar degeneration in later stages of the disease [41]. Limbic involvement is rare [43].

### 4.12 Anti-Yo

Anti-Yo, or purkinje cell autoantibody type 1, results in paraneoplastic cerebellar degeneration and only rarely can acute findings of FLAIR hyperintense signal change of the cerebellar hemispheres be seen [41]. The main cancer associations are breast and ovarian.

### 4.13 Anti-Ma2/Ta

MRI demonstrates abnormalities in up to 74% of patients with anti-Ma2 encephalitis including variable FLAIR hyperintense lesions of the diencephalon (thalamus/hypothalamus,) brainstem (midbrain, periaqueductal, pons, medulla oblongata, cerebellar peduncles), and limbic system (hippocampi) [44]. Furthermore, nodular tumor-like contrast enhancement has been described. Anti-Ma2 encephalitis is predominantly associated with testicular and lung tumors.

### 4.14 Anti-CV2

Typical findings of anti-CV2 encephalitis (associated with underlying small cell lung carcinoma or malignant thymoma) are characterized by FLAIR hyperintense signal change of the striatum and only rarely classic limbic encephalitis [36], [45].

### 4.15 Anti-GAD

Anti-GAD encephalitis is yet another antibody resulting in classic MRI findings of limbic encephalitis with FLAIR hyperintense signal change of the hippocampus and amygdala; the signal change is, in fact, significantly brighter than other forms of limbic encephalitis [46]. The hippocampal volume, however, was not significantly different, even when compared to control groups [47]. Long-term cerebellar degeneration has also been described [48].

This study has several limitations including the small number of cases which is expected with rare disorders. Second, while our study is single-center, we are a tertiary referral hospital and thus any patients with initial outside MRI imaging have been incorporated into our analysis. Third, no quantification of MRI abnormalities was performed. We feel, however, that the purely visual analysis most closely resembles clinical routine and allows for widespread impact, which was the aim of our study. Finally, the significance of the auto-antibody anti-VGKC has recently been disputed [49]. Due to the retrospective nature of our study and the fact that these patients received the diagnosis of anti-VGKC autoimmune encephalitis and corresponding treatment, they were included.

To conclude, we characterized the spectrum of initial, subacute, and long-term MRI findings in autoimmune encephalitis patients with 15 different autoantibodies and correlated these with treatment timepoints. Our MRI negative rate is comparable to what is found in the literature. There was a significant difference between the MRI spectrum of cell-surface vs intracellular antibody syndromes. Almost half of patients with initial/subacute findings demonstrated MRI improvement upon initiation of treatment. MRI can be used to help narrow the differential diagnosis in autoimmune encephalitis and can be used as a monitoring tool for certain subtypes of this rare disease.

## Ethics approval

Ethical approval was obtained through the Institutional Review Board (Kantonale Ethikkommission Zuerich, BASEC Nr. 2022–00041) prior to commencing the study.

## Informed consent

Informed consent was obtained for all patients included.

## Funding statement

This research received no specific grant from any funding agency in the public, commercial, or not-for-profit sectors.

## CRediT authorship contribution statement

**Michels Lars:** Writing – review & editing, Software, Resources, Methodology, Investigation, Formal analysis, Data curation. **Togni Claudio:** Writing – review & editing, Validation, Resources, Methodology, Investigation, Formal analysis, Data curation. **Hainc Nicolin:** Writing – original draft, Visualization, Supervision, Software, Resources, Project administration, Methodology, Investigation, Formal analysis, Data curation, Conceptualization. **Kulcsar Zsolt:** Writing – review & editing, Supervision, Software, Resources, Project administration, Methodology. **Simmen Cyril:** Writing – review & editing, Resources, Methodology, Formal analysis, Data curation. **Ludovichetti Riccardo:** Writing – review & editing, Software, Resources, Methodology, Investigation, Formal analysis, Data curation. **Nierobisch Nathalie:** Writing – original draft, Software, Resources, Methodology, Investigation, Formal analysis, Data curation, Conceptualization. **Abunada Mahmoud:** Writing – original draft, Resources, Project administration, Investigation, Formal analysis, Data curation, Conceptualization. **Terziev Robert:** Writing – review & editing, Resources, Methodology, Investigation, Formal analysis, Data curation, Conceptualization.

## Declaration of Competing Interest

The authors declare that they have no known competing financial interests or personal relationships that could have appeared to influence the work reported in this paper.
